# Green Protective Geopolymer Coatings: Interface Characterization, Modification and Life-Cycle Analysis

**DOI:** 10.3390/ma15113767

**Published:** 2022-05-25

**Authors:** Aoxuan Wang, Yuan Fang, Yingwu Zhou, Chenman Wang, Biqin Dong, Cheng Chen

**Affiliations:** 1 Key Laboratory for Resilient Infrastructures of Coastal Cities, Ministry of Education, Shenzhen University, Shenzhen 518060, China; 2017092097@email.szu.edu.cn (A.W.); yuanfang@szu.edu.cn (Y.F.); ywzhou@szu.edu.cn (Y.Z.); wangchm@szu.edu.cn (C.W.); incise@szu.edu.cn (B.D.); 2 Guangdong Provincial Key Laboratory of Durability for Marine Civil Engineering, College of Civil and Transportation Engineering, Shenzhen University, Shenzhen 518060, China

**Keywords:** geopolymer, protective coating, critical surface energy, modification, life-cycle analysis (LCA)

## Abstract

In the interest of solving the resource and environmental problems of the construction industry, low-carbon geopolymer coating ensures great durability and extends the service life of existing infrastructure. This paper presents a multidisciplinary assessment of the protective performance and environmental impacts of geopolymer coating. Various parameters, such as main substance, water-solid (W/S) ratio, activator type and curing time, were investigated for their effects on interface characterization in terms of contact angle, surface energy, mechanical properties and microstructure. These parameters had negligible effects on the amounts and types of hydrophilic functional groups of geopolymer surfaces. A combination of organic surface modifiers and geopolymer coatings was shown to ensure hydrophobic surface conditions and great durability. Silicon-based modifiers exhibited better wetting performance than capillary crystalline surfactants by eliminating hydroxyl groups and maintaining structural backbone Si-O-T (Si, Al) on geopolymers’ surfaces. Finally, life-cycle analysis was conducted to investigate the environmental performance. Geopolymer coating yielded substantially lower environmental impacts (50–80% lower in most impact categories) than ordinary Portland cement (OPC) coating. Silicon-based modifiers had negligible influence due to their minimal usage. Increasing the W/S ratio diluted the geopolymer coating and decreased the environmental impacts, and slag-based geopolymer coating achieved lower environmental impacts than FA-based and MK-based varietie.

## 1. Introduction

Economic growth and globalization necessitate for the growth of the construction industry. Global cement production experienced an annual 5% increase to 4.2 GT over the past 40 years, and is estimated to keep growing by 10% per year until 2060 [1]. A similar annual increase of 3.3% was observed in the steel industry [2]. To date, the carbon dioxide emission associated with the steel and concrete industry is responsible for 8% [3] and 6% [1] of global greenhouse gas emissions. Besides environmental pollution, the oversized construction industry also causes a shortage of natural resources and excessive solid waste. The construction industry is responsible for around 40–60% of the global consumption of natural resources, leading to the rising price of aggregate and sand [4]. Construction and demolition waste occupy around half of the municipal solid waste in China [5].

Reducing demand for the construction industry and decarbonation of construction material is essential for the sustainable development of civil infrastructure. Applying coatings on infrastructure efficiently reduces the need for new construction by prolonging the service life of existing structures. Deterioration in concrete structures is caused by a series of chemical and physical processes [6,7]. With its main consequence being chloride-ion-induced steel corrosion, sufficient and proper precautions are needed for offshore structures. Applying coatings has been proved efficient in inhibiting the entrance of harmful ions, thus reducing the resultant deterioration. 

Besides superior bond performance with concretes, most inorganic coatings possess adequate durability and great tolerance to high temperatures. However, the application of traditional cement-based coatings is restricted for various reasons. For instance, phosphate aluminate cement-based coating is renowned for its excellent durability due to is neutral pH values, yet the manufacturing process includes high-temperature calcination that emits a substantial amount of CO_2_ [8]. In fact, traditional cement-based coatings are all intensive in greenhouse gas emissions. Coatings of magnesium phosphate cement (mainly fabricated using KH_2_PO_4_, MgO, Borax) have the advantages of high adhesive strength, self-controlled shrinkages, great abrasive resistance. However, poor water resistance restrict their usage as protective coatings against corrosion [9]. Despite its extraordinary strength and tolerance to high temperature, magnesium oxychloride cement has a phase-transition-induced strength reduction when it encounters water, along with the possibility of chloride leaching [10]. Coatings using sulfur aluminate cement are vulnerable to carbonation due to low pH [11]. 

Geopolymer coatings are promising decarbonated alternatives to other cement-based counterparts. Synthesized mainly from industrial by-products (such as fly ash, slag and red mud), geopolymers excel in durability [12,13,14] with significantly reduced carbon dioxide emissions. Depending on their raw materials and activators [15], geopolymers mainly consist of calcium aluminosilicate hydrate (C-A-S-H) and sodium/potassium aluminosilicate hydrate (N/K-A-S-H). Thus, an absence of calcium hydroxide in the geopolymer matrix reduces expansive corrosion products and calcium leaching. In addition, the unique nature of N-A-S-H prevents the geopolymers from reacting with chloride and sulfur ions, thus maintaining its structural integrity [16]. Chloride prefers to be deposited on the resultant N-A-S-H by physical adsorption rather than chemical bonding, which prohibits further deterioration. Even under strong carbonation, different from Portland cement, the sodium carbonation and excessive sodium in the geopolymer system protects N-A-S-H from decomposition.

Therefore, the inherent durability of geopolymers and their superior bonding with cement-based materials make them desirable as protective coatings. In consideration of the hydrophilic feature of geopolymers, a combination of geopolymers and organic modifiers would satisfy both bonding and hydrophobic requirements. The interactions between the surfaces of various genera of geopolymer coatings (solid phase), especially those integrated with modifiers, and the liquid phase (water or water-containing corrosive components) need thorough study. Such interactions (coating and environment) can be characterized using surface energy and contact angle (CA) [17]. These two parameters are closely related to key properties of the coating, such as the strength of interactions, the stability of aqueous colloidal suspensions, and wetting, spreading and adhesion [17,18,19]. The traditional method for measuring contact angle is the sessile drop method [20]. However, such a method has strict requirements for the operating environment, including temperature, humidity and dust content. Any failure to meet the environmental requirements and to form a stable droplet on a surface of absorbing materials causes inaccuracies in the test results [21,22,23,24,25]. 

Furthermore, geopolymer coating has reduced environmental impacts attributed to decarbonation. As industrial waste products—such as slag—partially replace Portland cement in geopolymer, the overall usage of cement and the corresponding environmental impact potential is decreased. Geopolymer coating is thus considered more sustainable than other cement-based coatings. However, the exact environmental impact potentials of geopolymer coating have not been fully assessed. Moreover, existing studies tend to focus on the durability properties of geopolymer coatings and ignore the associated environmental performance. Hence, comparison of the environmental performances of geopolymer and traditional cement-based coatings is warranted. Without such an integrated assessment of protective and environmental performance, the optimization of geopolymer coating is biased.

In light of the summarized research insufficiency, this research aimed to design various geopolymer coatings based on the aggregated protective and environmental performance. Geopolymers were synthesized using slag, metakaolin, fly ash and red mud, solely or in combination with various water/solids ratios. Four different surface modifiers were also mixed in or applied on top of the geopolymer coating. The surface energy of the geopolymer was calculated based on the derivation of Thomas Young’s three-phase solid–liquid–gas equilibrium equation [26,27,28]. Moreover, the critical surface energy of geopolymers was obtained for geopolymer coatings in the water of different polar partial ratios, which can be utilized for predicting the wetting feature of a specific liquid on the surface of the geopolymer. The contact angle, surface energy and critical surface energy were used to identify and to explain the interface characterization of geopolymer coatings under various modifications. The wetting feature of a specific liquid on the surface of each geopolymer was described. A novel method—bubble method—was adopted for the measurement of the two-phase contact angle between geopolymer and deionized water. The environmental impacts of geopolymer coatings were comprehensively evaluated by life-cycle assessment (LCA). The environmental impacts of traditional cement-based coatings and geopolymer coatings were compared. The effects of the different mixtures on the overall environmental impacts were also discussed.

## 2. Materials and Methods

### 2.1. Materials

The geopolymer coatings were synthesized using slag, red mud, metakaolin and fly ash, whose chemical compositions are listed in Table 1. Two kinds of activators were used in this study: sodium water glass (molar ratio of SiO_2_:Na_2_O equal to 1.4 and its solids contents amounting to 39.76%, denoted as WG1.4 and NaOH (with a concentration of 10 mol/L or 14 mol/L). The alkaline contents, herein referred to as the mass fraction of Na_2_O, were 5% for all mixtures. The detailed mix proportions of geopolymer coatings are listed in Table 2. 

Specimen configurations were designed to consider the following parameters: (1) replacement of slag with raw materials—including red mud (RM), fly ash (FA) and metakaolin (MK)—at different replacement ratios (marked as group 1 in Table 2); (2) water-to-solid ratios varied from 0.3 to 0.6 (marked as group 2 in Table 2) in slag-based geopolymers; (3) partial replacement of slag with metakaolin at ratios ranging from 25% to 100% (marked as group 3 in Table 2); (4) various activators with the modulus of 1.4 and 2.0 (marked as group 4 in Table 2); and (5) different curing ages ranging from 28 days to a year (e.g., changes of the functional groups of surfaces), marked as group 5 in Table 2). Selected specimens were measured for contact angles and surface free energies in deionized water.

**Figure 1:**
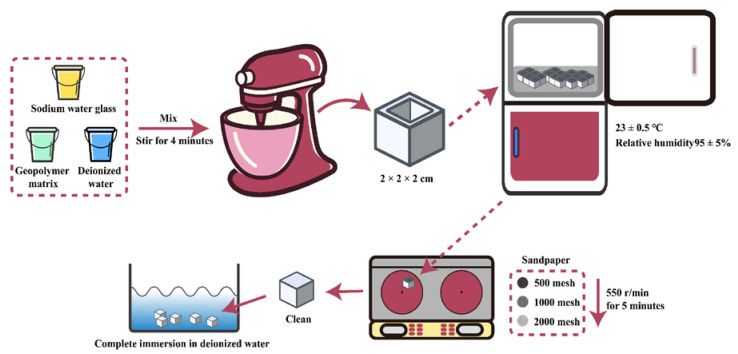
Schematics of geopolymers fabricating process.

The fabrication process is shown in Figure 1, and the polishing process is detailed in Section 2.3. The components were mixed for 4 minutes and vibrated for 2 minutes. All specimens were then cast in a steel mold (with dimensions of 2 cm × 2 cm × 2 cm), sealed with cling paper, and cured in an environmental chamber—with temperature 23 ± 0.5 °C and relative humidity 95 ± 5—for 28 days (except for group 5 in curing time) [29]. 

### 2.2. Surface Modification

To improve the surface characterization of geopolymer coating, four types of surface modifiers—methyl silicone oil (denoted as MSO, Guangzhou Xilong Chemical Co., Ltd., Guangzhou, China), nano organic silicon agent (denoted as OSA, Shanghai niuyuan industry and Trade Co., Ltd., Shanghai, China), capillary crystalline surfactants (denoted as SY, Xiya chemical industry and Trade Co., Ltd., Shanghai, China) and Compaktuna Pro Super (denoted as PTB, Polytechnisch Bedrijf, Shanghai, China)—were added to, or coated onto, the slag-based reference sample (W/S = 0.4). The produced samples are denoted by S0.4-MSO, S0.4-OSA, S0.4-SY and S0.4-PTB (Group 6 in Table 2), respectively. The addition method mixed PTB or SY into the geopolymer. The added weight of surface modifiers was chosen, ranging from 1% to 9% at an interval of 2%, and 5% had the best hydrophobic effect. The coated weight of MSO and OSA was 1% of the geopolymer coating. The contact angles of the modified geopolymer were then tested following the procedures described in Section 2.4. 

### 2.3. Surface Treatment 

The water contact angle was related to the types of functional groups on the material surface and the average roughness (Ra) of the material surface. In order to avoid the influence of Ra on the contact angle test, the sample was polished with sandpaper. The roughness coefficient of the treated surface can be considered as Ra = r = 1 [30]. All specimens were subjected to surface polishing with abrasive papers—500-mesh, 1000-mesh and 2000-mesh, sequentially—using a pre-milling machine. For each specification of abrasive paper, the sample was polished for 5 minutes at a speed of 550 r/min. Then the polished sample was rinsed and wiped using an ultrafine-fiber non-dust cloth to remove the dust and other impurities. After the sample was fully immersed in deionized water, preparation for subsequent testing of the surface contact angle was completed.

### 2.4. Measurement of Contact Angle 

Traditionally, the sessile drop method is applied to calculate the surface energy of solid materials. However, the testing process of the sessile drop method is greatly affected by temperature and humidity, leading to inaccurate measurement of contact angles [20,21,22,23,24,25]. The bubble method, by contrast, is insensitive to environmental disturbance [26,27,28], and was adopted herein to measure the contact angle of the geopolymer. Contact angles and surface properties of geopolymers were tested using an optical contacting angle instrument (OCA 20 model, manufactured by Data Physics Instruments, Filderstadt, Germany). Initially, the sample coated with the geopolymer was immersed in a transparent square water tank. A bubble of air or alkane was then released from a needle at a speed of 0.6 microliters per second, which was operated via the injection system of OCA instrument. Eleven types of alkane were used to produce the air bubbles; their chemical compositions and surface free energies are listed in [28]. Since the density of the chosen alkanes was lower than that of the water, the bubbles were well-formed and tightly attached to the test surface under the action of buoyancy. Moreover, as alkane is non-polar, all its surface energy comes from dispersion forces, which simplifies the boundary conditions. Deionized water was selected as the liquid phase, and its surface energy, polar and dispersion components were 72.8, 51.0, 21.8 mN/m [20]. A solid–water–alkane (air) three-phase system was formed after the bubble was perfectly attached to the surface. The contact angle between the alkane (air) and the solid surface was measured with the OCA instrument.

To ensure the accuracy of the test results, two points at different locations of the polished surface of each sample were selected, and the arithmetic mean values of the left contact angle and right contact angle were calculated. For each mix proportion of the geopolymer, three samples were tested. Throughout the test process, powder-free gloves were worn to reduce the contamination of samples. When not being tested, the samples were immersed in deionized water to avoid carbonation. 

### 2.5. Surface Free Energy, Work of Adhesion and Critical Surface Energy

As an important indicator of wetting degree in a solid–liquid system, the work of adhesion is related to the surface energy of two contacted phases [28,31]:

(1) $$W_{a}=\gamma_{1}+\gamma_{2}-\gamma_{12}$$

where *W_a_* is the work of adhesion, *γ*_1_ and *γ*_2_ are the surface energy of phase 1 and 2, respectively, and *γ*_12_ is the interfacial energy between phase 1 and phase 2.

Surface energy is the action resulting from different molecular forces, among which the dispersion force and polarity force are the most important [25,28]. Therefore, the total surface energy and work of adhesion can be divided into a dispersion-force component and a polarity-force component [25,26,28,32,33]:

(2) $$\gamma =\gamma^{d}+\gamma^{p}$$

(3) $$W_{a}=W_{a}^{d}+W_{a}^{p}$$

(4) $$W_{a}=2\cdot \sqrt{\gamma_{1}^{d}\cdot \gamma_{2}^{d}}+2\cdot \sqrt{\gamma_{1}^{p}\cdot \gamma_{2}^{p}}$$

where *γ* is the total surface energy, *γ_d_* and *γ^p^* are the dispersive-force and polarity-force components and *W_a_^d^* and *W_a_^p^* are the work of adhesion contributed by the dispersion force and polarity force between molecules. 

In the solid–liquid–gas system, the liquid forms a certain contact angle (0°–180°) on the solid surface and reaches an equilibrium. For a low-energy surface, it satisfies the Young equation [12]:

(5) $$\gamma_{sw}=\gamma_{si}-\gamma_{wi}\cos\left(\theta \right)$$

where *γ_sw_*, *γ_si_* and *γ_wi_* are the solid–water, solid–alkane, and water–alkane interfacial surface tensions, respectively; and θ refers to the equilibrium contact angle or eigen contact angle of the material. Wetting only occurs when θ is smaller than 90°, and a smaller contact degree indicates better wettability of the liquid [34]. N-alkane was adopted in this study, and its surface energy was solely composed of the dispersion component.

From Equations (1), (4) and (5), the surface energy of the solid can be estimated using the following equation:

(6) $$\gamma_{l}\left(1+\cos\left(\theta \right)\right)=2\cdot \sqrt{\gamma_{s}^{d}\cdot \gamma_{l}^{d}}+2\cdot \sqrt{\gamma_{s}^{p}\cdot \gamma_{l}^{p}}$$

where $\gamma_{l}$ denotes the surface energy of the liquid, $\gamma_{s}^{d}$ and $\gamma_{s}^{p}$ are the dispersion-force component and polarity-force component of the surface energy of the solid and $\gamma_{l}^{d}$ and $\gamma_{l}^{p}$ are the dispersion-force component and polarity-force component of the surface energy.

When the three-phase solid–water–alkane (air) system reaches equilibrium, the surface energy of any two-phase system (solid–water phase, solid–alkane/air phase, or water–alkane/air phase) must satisfy Young’s equation [12]. Substituting the surface energy for solid–water, solid–alkane and alkane–water into Equations (1), (4) and (5), the surface-energy calculation based on the bubble method is obtained:

(7) $$\frac{\gamma_{w}+\gamma_{wi}\cos\left(\theta \right)-\gamma_{i}}{2\cdot \sqrt{\gamma_{w}^{p}}}=\sqrt{\gamma_{s}^{d}}\cdot \frac{\sqrt{\gamma_{w}^{d}}-\sqrt{\gamma_{i}}}{\sqrt{\gamma_{w}^{p}}}+\sqrt{\gamma_{s}^{p}}$$

The solid dispersion and polarity components can be obtained from Equation (7), and a linear expression in *x* and *y* can be obtained through a linear fitting, namely, $y=\sqrt{\gamma_{s}^{d}}\cdot x+\sqrt{\gamma_{s}^{p}}$. Here, the square of the slope is the dispersion component of the solid surface energy, and the square of the intercept is the polarity component of the solid surface energy; the sum of both is the sum of the sample’s surface energy. 

From Equation (6), the contact angle can be expressed as a function of critical surface energy [25,28,35]:

(8) $$\cos\left(\theta \right)=\frac{2\sqrt{\gamma_{l}^{d}\cdot \gamma_{s}^{d}}}{\gamma_{l}}+\frac{2\sqrt{\gamma_{l}^{p}\cdot \gamma_{s}^{p}}}{\gamma_{l}}-1$$

In the cases that θ equals zero (i.e., the surface-free energy of a liquid equals the critical surface energy of the solid), Equation (8) can be further simplified as:

(9) $$\gamma_{c}=\gamma_{l}={\left[\sqrt{\left(1-\omega_{l}^{p}\right)\cdot \left(1-\omega_{s}^{p}\right)}+\sqrt{\omega_{l}^{p}\cdot \omega_{s}^{p}}\right]}^{2}\cdot \gamma_{s}$$

where $\omega_{l}^{p}$ is the polarity-force component of the liquid surface energy, and the dispersion-force component of the liquid surface energy is expressed as 1 − $\omega_{l}^{p}$; $\omega_{s}^{p}$ is the polarity-force component of the solid surface energy, and the dispersion-force component of the solid surface energy is expressed as 1 − $\omega_{s}^{p}$; $\gamma_{c}$ is the critical surface tension, a characteristic of solid surface. The critical surface energy and the dispersion-force components can be calculated [28,36] using Equation (9).

### 2.6. Mechanical Property 

**Figure 2:**
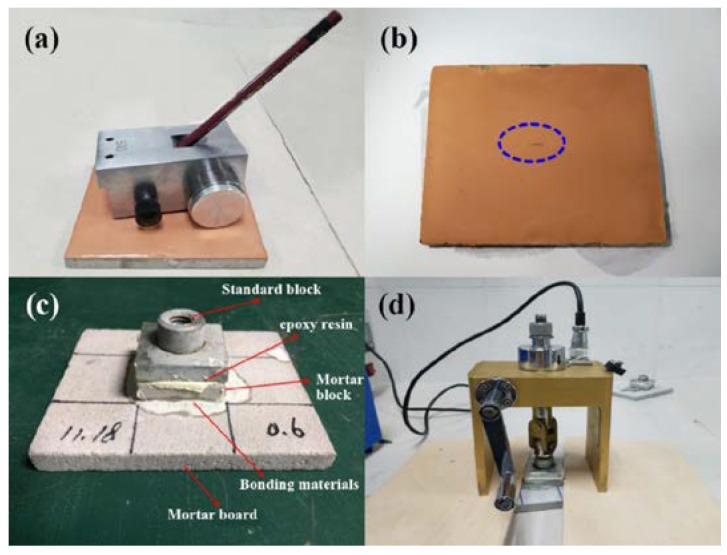
Testing regime and diagram for hardness and adhesive strength of geopolymers: (**a**) hardness testing kit; (**b**) scratched surface of geopolymer coating; (**c**) setup for adhesive strength testing; and (**d**) testing kit for adhesive strength testing.

Hardness testing and adhesive strength testing were performed. For the hardness test, the designed kit in Figure 2a was used with 20 pencils, with hardness ranging from 9H-H to B-6B based on the Brinell hardness classifications in accordance with standard GB/T6739-1996. After the application of force using the designed kit without bending the 3 mm pencil core, the surface should not have obvious scratches, as in Figure 2b. To evaluate each sample’s adhesive strength with Portland cement, tests were performed as per standard JGJ110-2008. The standard block was made of 45-gauge steel with the size of 40 mm × 40 mm × (6–8) mm, the binder was epoxy resin, the mortar block and the substrate material was cement mortar and the bond layer was ground poly coating. The specimen was fabricated as shown in Figure 2c. The instrument is intelligent bond strength tester (XH-6000N, Hebei, China) and test set-ups were demonstrated in Figure 2d. After the instrument was installed, it was loaded at 20 N/s until the geopolymer coating was peeled off from the cement mortar board, and the data were recorded. The obtained adhesive strength was an average result of three specimens. 

### 2.7. Functional Group and Microstructure

An FT-IR coupled with a microscope (Frontier MIR, Spotlight 400, Waltham, MA, USA) was used to collect functional group vibrations in the 4000–500 cm^–1^ range of the geopolymer surfaces. After the coatings were immersed in deionized water for 90 days, surface morphology observation was conducted using a trinocular stereo-microscope (XTL-3000C, Caikon, Shanghai, China). Selected samples based on their stereo-microscope results were denoted as Reference-S0.4-90 and S0.4-MSO-90, respectively.

## 3. Results and Discussion

Detailed results of the tests described in the previous context are presented in this section. Appropriate discussions were also conducted to identify the factors that affected the test results.

### 3.1. Contact Angle, Surface Energy and Work of Adhesion

The surface characteristics of coating are essential to understand the interface interaction between coating and environment. As per classic thermodynamics, wetting occurs when the surface energy of the coating-environment system is reduced under constant temperature and isobaric pressure. The extent of surface-energy reduction determines the extent of wetting, which is indicated by the work of adhesion. In a solid–liquid system, the larger work of adhesion means a greater wetting degree. The derivation of surface energy and work of adhesion from the contact angle was detailed in Section 2.6.

**Figure 3:**
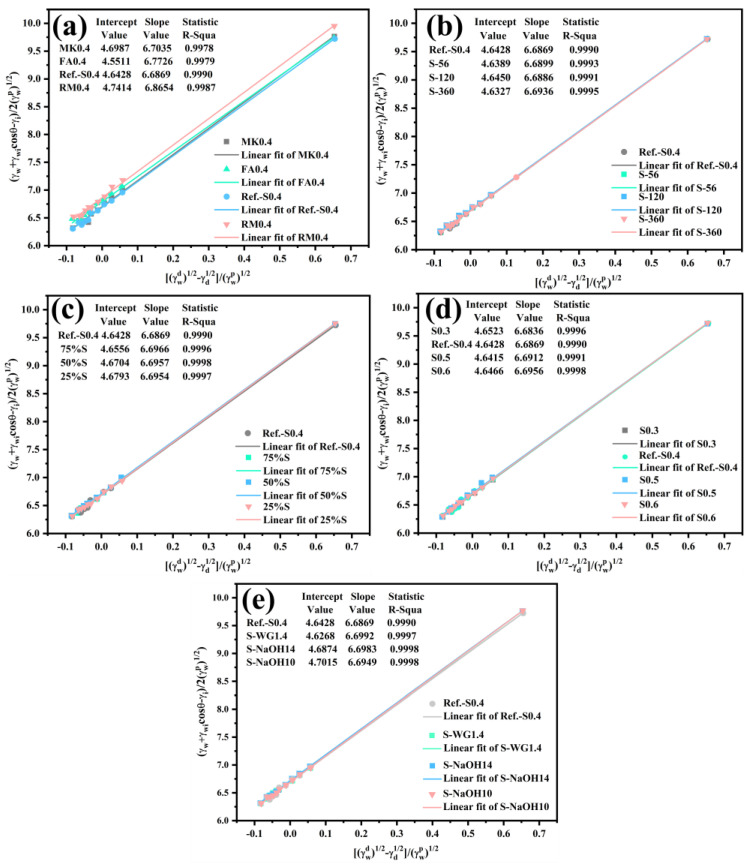
CA linear fitting of geopolymers: (**a**) various w/s ratios; (**b**) different ages; (**c**) blends of MK and slag; (**d**) various raw materials; (**e**) various activators.

Based on Equation (7), Figure 3 was plotted to calculate the surface energy and adhesion work (the CAs of geopolymers activated by water glass or NaOH are given in the Appendix A Table A1). It can be seen that the variables $\left(\sqrt{\gamma_{w}^{d}}-\sqrt{\gamma_{i\;}}\right)/\sqrt{\gamma_{w}^{p}}$ were linearly proportional to ($\gamma_{w}+\gamma_{wi}\cos\left(\theta \right)-\gamma_{i}$)/$\;2\cdot \sqrt{\gamma_{w}^{p}}$ with R-square value over 0.9995 (see Figure 3). The surface energy and the work of adhesion were calculated and listed in Table 3. The variation in water content, represented by water to solid ratios (W/S), showed negligible effect on the surface energy of geopolymer coatings and on the wetting performance, represented by the work of adhesion. Regardless of the W/S ratios, the components of the work of adhesion were distributed within reasonably small ranges in Figure 3a. For instance, the dispersion component values were distributed in the range of 21.54–21.64 mN/m, whereas the polarity components were in the range of 44.67–44.83 mN/m. The surface energies were distributed in the range of 66.28–66.42 mN/m and the work of adhesion values were distributed in the range of 138.87–139.02 mN/m. As reported in previous research by the authors [28], however, the surface energy of traditional cement-based coating is affected by the water content. For instance, the surface energy of OPC was distributed in the range of 67.74–74.58 mN/m (RFE), higher than the 66.26–69.61 mM/m for all unmodified geopolymers (Table 3). Hence, the surface energy of OPC was more prone to water than geopolymers. The difference between the surface energy of OPC and geopolymer coatings can be explained by distinctive reaction mechanisms. The hydration reaction of OPC is mainly the hydration of calcium silicates, resulting in C-S-H and calcium hydroxide, etc. [37]. The hardening process of geopolymer mainly involves the hydrolysis of silicate and aluminate, which forms a three-dimensional network polymer constituted of silica tetrahedron and alumina tetrahedron. Given the abundant calcium in the raw material, C-S-H may also be formed in geopolymer [38,39]. The terminal functional groups of OPC and geopolymers are both hydrophilic hydroxyl groups, and the number of hydroxyl groups may affect the surface properties. 

Due to the unsatisfactory reactivity of red mud, the strength of geopolymer coating using red mud was too low for application as a protective coating. Geopolymer coating using red mud also had the largest work of adhesion of 142.33 mN/m, which was attributed to the poorly reacted red mud and its relatively abundant hydroxyl groups (see Table 3). As the poorly crystalline red mud has a much higher pH value, its surface is usually condensed with an unneglectable amount of hydroxy sodalite [40].

In summary, the amount of water (the water/solids ratio ranges from 0.3 to 0.6), the properties of activators (water glass and NaOH), the constitutions of raw materials (except for the red mud) and ages for curing have a negligible effect on the amounts and types of hydrophilic functional groups in geopolymer surfaces. Red mud has relatively unsatisfactory reactivity, and heat treatment is required for purely fly ash and metakaolin-based geopolymers, curbing their mechanical performance. A reference specimen was selected for the following modifications, as discussed below. 

### 3.2. Surface Properties, Functional Groups and Microstructures after Modification

In this section, four surface modifiers were applied to the reference specimen to investigate the effects on the surface properties and microstructures.

#### 3.2.1. Surface Properties 

**Figure 4:**
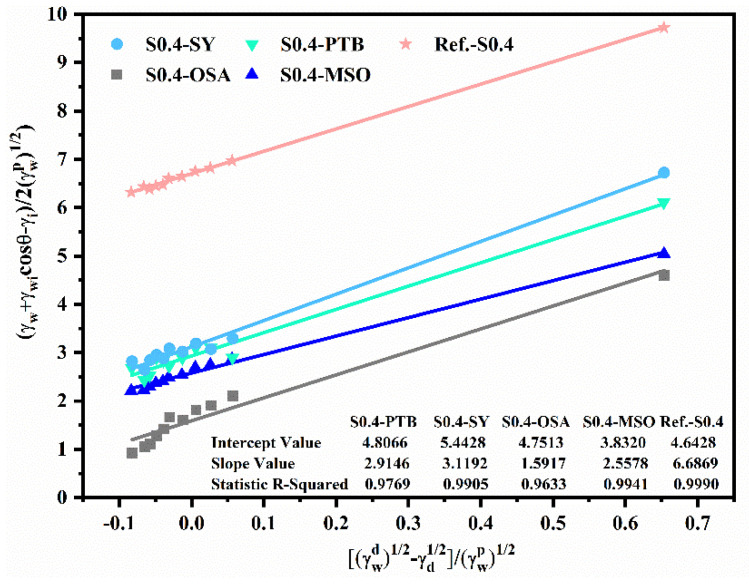
CA linear fitting of geopolymers after surface modification.

Surface modifications were used to investigate their effect on the geopolymer wetting performance. The detailed configurations were described in Section 2.2 and Table 2, and the measured contact angles of geopolymer after modification are listed in Appendix A Table A2. Figure 4 shows the contact angles of geopolymers with various surface modifiers and with eleven alkane media and the air. The contact angle greatly increased after modification as compared to the reference specimen. The solid–liquid contact angle with air as bubbles of Reference-S0.4, S0.4-SY, S0.4-PTB, S0.4-MSO and S0.4-OSA increased from 24.93° to 95.63°, indicating the surface of geopolymer coating was changed from hydrophilic to hydrophobic. This can also be identified via the surface free energies: hydrophilic ranged from 50 to 100 mN/m, and hydrophobic from 0 to 35 mN/m [23]. However, contact angles of geopolymer coatings mixed with crystalline surfactants (specimens S0.4-SY and S0.4-PTB) were evidently smaller than those coated with MSO and OSA. This was because of the different working mechanisms: crystalline surfactants functioned as pore-structure refiners by constantly reacting with calcium-rich compounds and forming crystalline fillers [41,42]; the MSO and OSA replied upon the strongly hydrophobic functional groups—the R-groups—to firmly bond with the terminal hydroxyl groups exposed on the surface of geopolymers to repel water. The latter mechanism proved more effective in this study. 

The effect of surface modifier on surface energy and work of adhesion is shown in Table 4. The work of adhesion for each of the samples S0.4-PTB, S0.4-SY, S0.4-OSA and S0.4-MSO, calculated using Equation (4), was 37.7%, 31.3%, 51.7% and 47.9% less than that of Reference-S0.4, respectively. These results also indicated better hydrophobic properties after surface modification.

#### 3.2.2. Evolution of Functional Groups and Microstructures 

**Figure 5:**
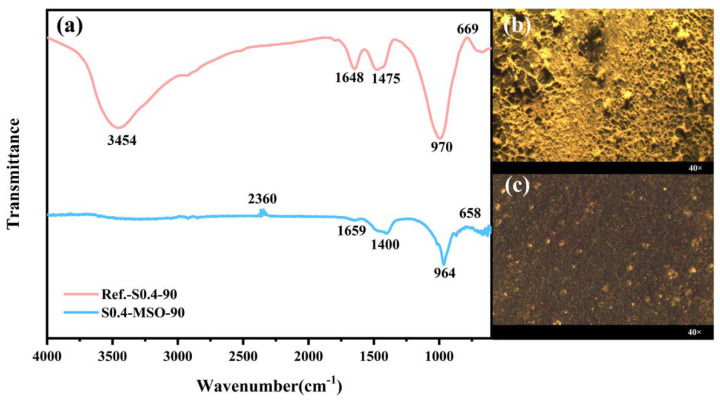
(**a**) The surface FTIR spectra and their stereo-microscope images; (**b**) Reference-S0.4-90 and (**c**) S0.4-MSO-90.

**Figure 6:**
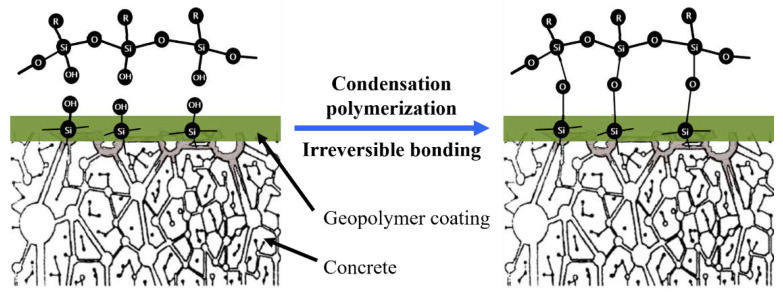
Diagrammatic sketch of bonding process between MSO and geopolymer coating.

To further reveal the mechanism of surface modification, the surface functional group evolution in the geopolymer coatings were studied. Based on the experimental results of the contact angle in the previous section, samples S0.4-MSO-90 and Reference-S0.4-90 were selected and immersed in water for 90 days for nondestructive IR-microscope and stereo-microscope testing. The results are shown in Figure 5. The peaks at 3454 cm^−1^ and 1648 cm^−1^ proved the presence of hydroxyl groups in the geopolymer matrix [43], which were obviously weakened or even disappeared after modification. The vibrational band at 1475 cm^−1^ attributed to $v_{3}$[CO_3_^2−^] and the Si–O vibration band generated by the SiO_4_ groups in the anhydrous slag shifted from 996 cm^−1^ to 961–969 cm^−1^ due to the formation of calcium aluminosilicate hydrate (C-A-S-H) [44,45]. The signal at 658–669 cm^−1^ was due to the stretching vibrations generated by the Al–O bonds in the AlO_4_ groups [44,46]. Results suggested that the main matrix of geopolymers (i.e., the linkages of the silicon-oxygen tetrahedron and aluminum tetrahedron) was maintained after modification. Nevertheless, the visible hydrophobic condition of geopolymer surfaces and the absence of hydroxyl groups were observed. Figure 6 shows the diagrammatic sketch of this mechanism: the active functional groups of silicone modifiers, e.g., H, OR, OH, etc., attracted surface-active groups and adsorbed water to form a firm hydrogen bond to the substrate surface. The active functional groups also aligned the non-polar organic groups to form a hydrophobic film [47]. 

After 90-day immersion in water, pores measuring 0.03–0.08 mm in diameter appeared in Reference-S0.4-90, as shown in Figure 5b, acting as conductive channels for the diffusion of chloride ions. In other words, the coating deteriorated during the accelerated exposure to chlorides [48]. Furthermore, the white deposits in Figure 5b were the product of alkali precipitation. In contrast, the surface of the modified coating S0.4-MSO-90 in Figure 5c was denser and more compact. Due to the hydrophobic effect, alkali precipitation in the geopolymer was mostly prevented. In this regard, the MSO modifier improved the durability of geopolymer coating. 

### 3.3. Physical Properties 

**Figure 7:**
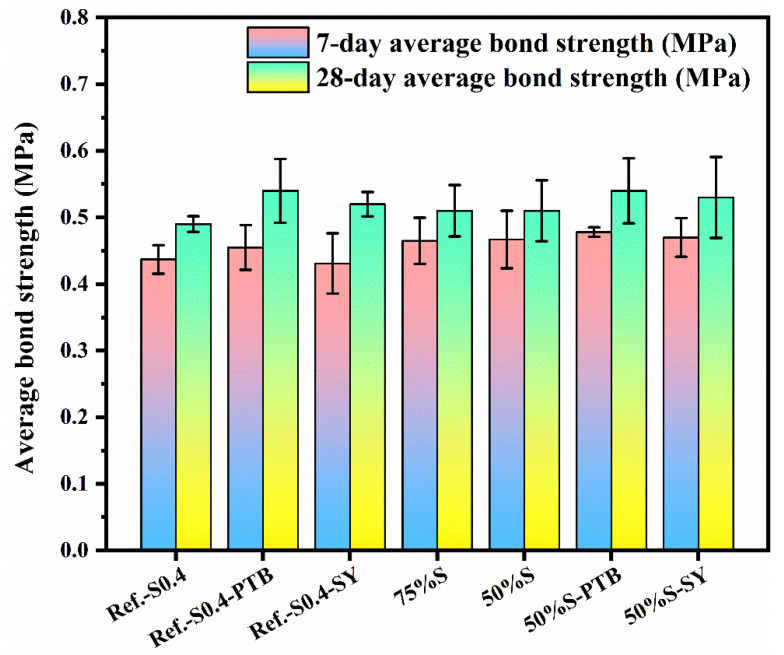
Adhesive strengths of various geopolymer coatings with cement paste.

In the hardness test, none of the coatings had obvious scratches, as with the results in Figure 2b. As the surface modifiers OSA and MSO had a negligible effect on the adhesive strengths with OPC, the resulting specimens are thus excluded hereafter. The adhesive strengths of geopolymers with OPC are shown in Figure 7. 

All the specimens exhibited similar adhesive strengths at the same age. The added modifiers PTB and SY marginally enhanced the adhesive strengths. The addition of PTB and SY eliminated hydroxyl groups, visibly increasing the capacity for preventing water from entering the interior, hence refining the pore structures of the resulting pastes, which was also confirmed by all elevated strengths at 28 days.

The replacement of slag by metakaolin also refines pore structures, but metakaolin works in a different manner: the metakaolin particles filled mesopores to increase bond strength, as demonstrated by 50%S and 75%S in Figure 7. No noticeable difference existed between the bond strength of 50%S and 75%S. The slow release of active silicon and aluminate in metakaolin resulted in the early-stage polymerization being dominated by the hydration of slag. Due to the abundant calcium in slag (Table 2), the main reaction products during this stage were C-A-S-H. The absence of calcium in metakaolin and lack of heat curing, meanwhile, restricted further strength development in geopolymer coatings using metakaolin. 

### 3.4. Critical Surface Energy

**Figure 8:**
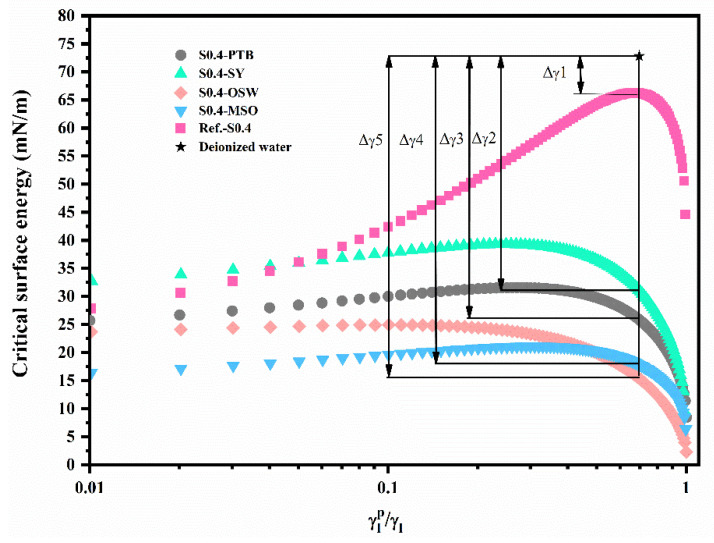
The critical surface-energy curve of geopolymers after the modification.

Besides providing basic surface-energy information about the material, the critical surface-energy curve identifies the hydrophobic properties of geopolymer coating in certain liquids that cannot be determined through the work of adhesion [28]. For example, the hydrophobic properties of geopolymer in NaCl solution or sulfate solution cannot be obtained using the work of adhesion. The derivation of critical surface energy and the dispersion-force components are detailed in Section 2.5, and the critical surface-energy curve is illustrated in Figure 8. The surface energy of deionized water is located above the curve, indicating a contact angle existed between deionized water and the surface of geopolymers. The intercept of the critical surface-energy curve at the customary vertical axis, $\omega_{p}$ = 0, is the dispersion component of the material, and the intercept when the vertical axis is taken to be $\omega_{p}$ = 1 is the polarity component of the material; the peak value of the curve represents the total surface energy of the material. For Reference-S0.4, the maximum critical surface energy was 66.28 mN/m, and the critical surface energy when $\omega_{p}$ equaled 1 was around 44.72 mN/m, which was the polarity component. 

The wettability of the specimens, represented by energy difference, could also be identified from the critical surface-energy curves. The energy difference (Δγ) was the difference between the surface free energy of liquid and the critical surface energy of the solid. Figure 8 shows the energy difference for all five samples, and the values of Δγ were in the order $\Delta \gamma_{1}<\Delta \gamma_{2}<\Delta \gamma_{3}<\Delta \gamma_{4}\approx \Delta \gamma_{5}$. Substituting Equation (8) into Equation (9), the relationship between the critical surface energy and contact angle can be derived:

(10) $$\cos\left(\theta \right)=2\sqrt{\frac{\gamma_{c}}{\gamma_{l}}}-1$$

Substituting $\gamma_{c}=\gamma_{l}-\Delta \gamma$ into Equation (10), the contact angle can be expressed in terms of energy difference:

(11) $$\cos\left(\theta \right)=2\sqrt{\frac{\gamma_{l}-\Delta \gamma }{\gamma_{l}}}-1$$

Therefore, a larger energy difference indicates a larger contact angle and better hydrophobic properties. Obviously, the samples S0.4-OSA and S0.4-MSO, having similar energy difference ($\Delta \gamma_{4}\approx \Delta \gamma_{5}$), had better hydrophobic properties than those of S0.4-PTB and S0.4-SY, which was consistent with the results based on the work of adhesion. 

## 4. Environmental Impacts

Life-cycle assessment (LCA) is a powerful tool to evaluate the environmental impacts associated with a product. With appropriate life-cycle inventories and system boundaries, LCA generates accurate estimates of potential environmental impacts in different aspects. Though still in early-stage development, LCA can identify the main contributors of environmental impacts and assist in decision-making by pointing out potential aspects for future improvement. 

A complete and valid framework of LCA generally comprises four major sections in accordance with the definitions described in the international ISO standards 14040 and some highly cited articles [49,50], namely, the goal and scope, life-cycle inventory analysis, life-cycle impact assessment and life-cycle interpretation. These are discussed in detail as follows.

### 4.1. Goal and Scope

Though geopolymer is widely accepted as an eco-friendly alternative to traditional coating, its environmental performance has not been thoroughly investigated. An LCA was conducted in this work to establish a direct comparison between the environmental performance of ordinary Portland cement (OPC) and geopolymer coatings. Furthermore, the effects due to various parameters—such as surface modifier, mixture, substitution ratio of slag with MK, and W/S ratio—on the overall environmental performance were also analyzed and displayed. The total potential and the specific contributions of each substance were obtained and presented in each subplot. 

**Figure 9:**
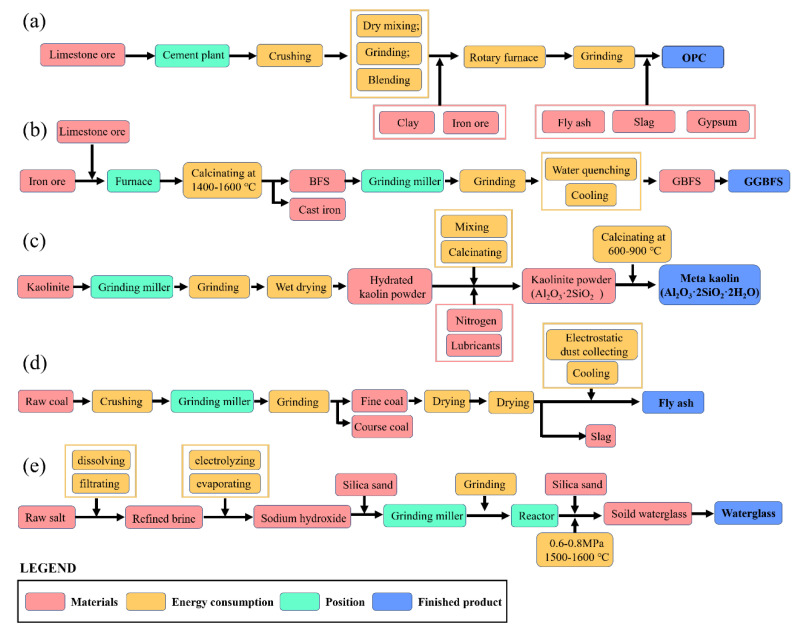
Life-cycle boundary of: (**a**) OPC; (**b**) slag; (**c**) MK; (**d**) FA; (**e**) water glass.

Among all the considered specimens, five materials were used in the mixtures of geopolymer coatings: slag, MK, FA, water glass and water. OPC served as the benchmark, and OSA was the only organic coating. Figure 9a shows the system boundary for OPC coating. Ground granulated blast furnace slag (GGBFS) was used in this study; its system boundary, shown in Figure 9b, starts with blast furnace slag (BFS) from pig iron production. The BFS is then ground to produce granulated blast furnace slag (GBFS). GBFS usually goes through drying, crushing, grinding and storage before it is transformed to GGBFS. As displayed in Figure 9c, the system boundary of MK originates from the mining of kaolin. To separate kaolin from other substances, such as silica sand, a wet processing method is applied to transform kaolin into the slurry. After passing through classifiers, centrifugation, separation, flotation, drying and grinding, the slurry is processed to become purified kaolin powder. MK is finally produced by the heating of kaolin to 570-600 Celsius degrees in a rotary furnace. FA is a side product of coal combustion, as shown in Figure 9d, going on to be captured by filter bags, transferred to storage and packed for sales. Transportation distance of FA is taken as 100 km. Water glass, or sodium silicate, is produced by mixing pure sand with alkali carbonate in Figure 9e. The mix is fed into a furnace and heated to 1600 °C. During the heating process, silicon dioxide in silica and sodium carbonate in soda are transferred to sodium silicate. The sodium silicate is then ground to powder. Ion exchange is modeled to produce deionized water, and cations and anions are exchanged with protons and hydroxide ions. 

**Figure 10:**
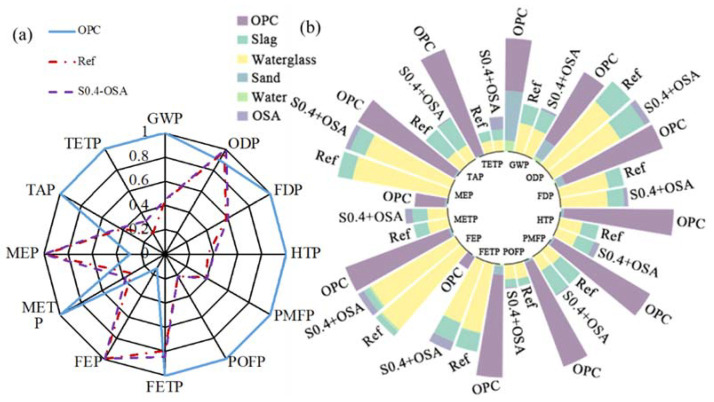
LCA results due to coating type: (**a**) summary; (**b**) substance contribution.

**Figure 11:**
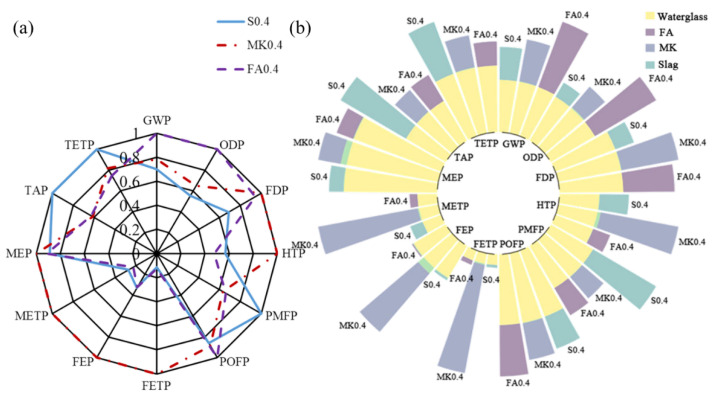
LCA results due to slag, FA and MK: (**a**) summary; and (**b**) substance contribution.

**Figure 12:**
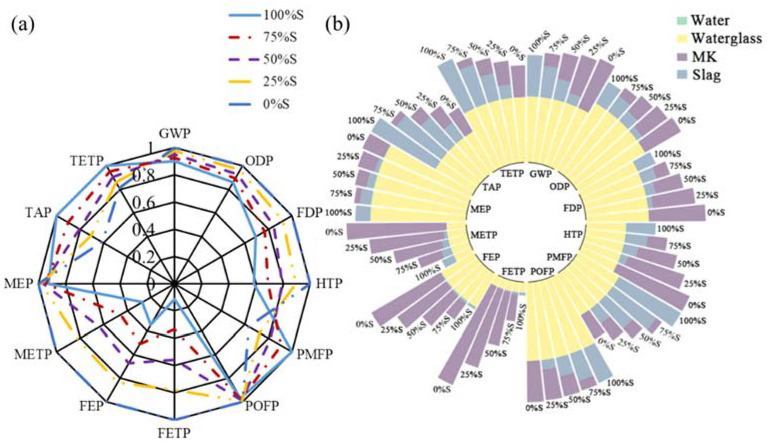
LCA results due to the mix of slag and MK: (**a**) summary; and (**b**) substance contribution.

**Figure 13:**
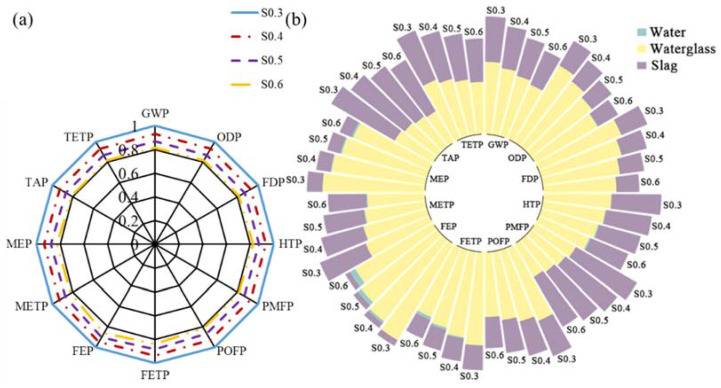
LCA results due to W/S ratio: (**a**) summary; (**b**) substance contribution.

The “functional unit” (FU) is defined as coating of equal volume, i.e., 1 m^3^ herein. For the comparison between geopolymer and Portland cement coatings, specimens Reference and S0.4-OSA were selected.in Figure 10 To illustrate the effect due to the main substance (i.e., slag, MK and FA) of geopolymer coating, the environmental performance of specimens S0.4 (sample Reference), MK0.4 and FA0.4 were represented in Figure 11. All three specimens had the same w/d ratio of 0.4; the mass fraction of water glass and water were also consistent among all specimens. As MK functions as a pore refiner in the short term and develops strength in the long term, the substitution ratio of slag with MK was also studied based on the environmental performance of specimens 100%S (sample Reference), 75%S, 50%S, 20%S and 0%S, as shown in Figure 12. All five specimens had an identical mass fraction of water glass and water, whereas the mass ratio between slag and MK varied from 0 to 1. Finally, the effect due to the W/S ratio on the environmental performance of geopolymer coating is investigated. Four specimens—S0.3, S0.4 (sample Reference), S0.5 and S0.6—were considered for investigation, shown in Figure 13.

To evaluate the environmental performance, an approach of the cradle-to-gate genre of LCA, involving a sector life beginning with raw material acquisition and ending with the completion of the production process, is adopted in this research. Due to the insignificant environmental impact within the use stage and the complication in quantification, this type of LCA is recommended.

### 4.2. Life-Cycle Inventory Analysis

The precision of LCA is restricted by the availability and quality of background data. Any lack of data concerning one or several processes within material flows would lead to inaccurate LCA results. 

For the considered life-cycle boundary in Figure 9, the life-cycle inventory of all substances is listed in Table 5. All background data were from two sources: Gabi dataset [51] and Ecoinvent dataset [52], and the corresponding environmental impacts were calculated using Gabi professional. Some materials adopted aggregate processes in Gabi dataset, such as OPC, FA, MK, OSA and deionized water. The Ecoinvent dataset was mainly used for slag and water glass. 

### 4.3. Life-Cycle Impact Assessment 

In this section, all life-cycle inventory from the previous section is substituted into Gabi Professional software using the ReCiPe midpoint method (www.lcia-recipe.net, accessed on 4 June 2021) to generate life-cycle impact assessment. The ReCiPe midpoint method comprehensively evaluates the environmental impact with twelve indicators, i.e. global warming potential (GWP), ozone depletion potential (ODP), fossil depletion potential (FDP), human toxicity potential (HTP), particulate matter formation potential (PMFP), photochemical oxidant formation potential (POFP), freshwater eutrophication potential (FEP), freshwater ecotoxicity potential (FETP), marine eutrophication potential (MEP), marine ecotoxicity potential (METP), terrestrial acidification potential (TAP) and terrestrial ecotoxicity potential (TETP). In other words, twelve indicators—instead of a total one—are given as results of life-cycle impact assessment. Each indicator considers contributions from different sources. For instance, the unit of GWP is the weight of carbon dioxide, and all the other greenhouse gases (GHG) are equivalently transferred to carbon dioxide based on their respective impact intensity on global warming. Similar treatment is adopted by the other eleven indicators. The twelve indicators comprehensively cover the environmental impact on the human body, sustainability, environment, etc. For instance, FEP, FETP, MEP and METP focus on the results of human activities on the water body, and GWP, ODP and FDP are associated with consequences on climate change and energy consumption. Besides the total potential, the contributions of different constituents to each potential are also calculated to facilitate the identification of main contributors.

### 4.4. Life-Cycle Assessment Interpretation

The following environmental impacts are calculated based on the respective FU. Figure 10a, Figure 11a, Figure 12a and Figure 13a show the general results of potential intensity (PI), which normalizes each environmental potential by the largest potential of six specimens:

(12) $$PI_{i,j}=\frac{P_{i,j}}{\max\left(P_{1,j},\;P_{2,j},\;P_{3,j},\;\ldots \right)}$$

where *P_i,j_* = environmental impact potential of the *i*th specimen in the *j*th indicator, and *PI_i,j_* = potential intensity of the *i*th specimen in the *j*th indicator. 

To identify the contribution of each substance, Figure 10b, Figure 11b, Figure 12b and Figure 13b decompose the total potential intensity of individual specimens to the contributions of all involved substances.

#### 4.4.1. General Results

Figure 10a directly compares the environmental performance of OPC coating, geopolymer coating and OSA coating on top of geopolymer coating. With eight out of twelve indicators (GWP, FDP, HTP, PMFP, POFP, FETP, METP, TAP and TETP), a pronounced decrease in environmental potentials was established by replacing OPC coating with geopolymer coating. All coatings had similar impact potential in ODP, and geopolymer coating yielded higher potential than OPC coating in FEP and MEP. Therefore, geopolymer coating was significantly more eco-friendly than traditional OPC coating. The effect of OSA was negligible in most indicators (except for FETP and FEP) due to its low weight fraction (i.e., 1%). Considering the increase in contact angle by OSA, OSA coating on top of geopolymer coating yielded optimum hydrophobic and environmental performance.

Figure 11a compares the effect of FA, MK and slag on the environmental performance of geopolymer coating; the reference sample is denoted by S0.4 herein. Among the twelve indicators, the geopolymer coating using MK reached the highest environmental impact potential with six, i.e., FDP, HTP, FETP, FEP, METP and MEP. Meanwhile, geopolymer coating using FA and slag each yielded the highest potential with three indicators. The environmental burden on the water system and human health was significantly relieved when slag or FA was used for geopolymer coating instead of MK. Hence, the environmental performance of FA- and slag-based geopolymer coating was similar, both superior to the MK-based coating.

Figure 12a assesses the environmental performance of geopolymer coating mixed with slag and MK, the reference sample is denoted by 100%S herein. The total weight of slag and MK remained constant, and 25%, 50%, 75% and 100% MK was replaced by equal-weight slag. When slag replaced MK, an obvious decrease in impact potential was observed in the indicators of FDP, HTP, PMFP, FETP, FEP, METP, and TAP. Meanwhile, the change for the GWP, ODP, POFP and MEP indicators was negligible. In other words, the replacement of MK with slag lowered the environmental impacts of geopolymer coatings. As the hydrophobic properties of MK- and slag-based geopolymer coatings were similar, slag-based geopolymer is recommended because of its low environmental impacts.

Figure 13a assesses the environmental performance of geopolymer due to the W/S ratio; the reference sample is denoted by S0.4 herein. The weight of slag and water glass remained constant, and the adjustment of the W/S ratio was fulfilled by the addition of different amounts of deionized water. As the environmental impact of deionized water was negligible compared to the other substances—i.e., slag and water glass—in the geopolymer coating, increasing the W/S ratio effectively decreased environmental impact potentials.

#### 4.4.2. Discussion

Sand and OPC are two major contributors to the environmental impacts of OPC coating, as shown in Figure 10b. In coatings using geopolymer, water glass contributes considerably to the overall environmental impact potentials. As the production of water glass required heating at high temperature and emitted heavy metal ions into the water environment, the ODP, FEP and MEP of water glass in Figure 10b—and the total geopolymer coating in Figure 10a—were thus high. The heating and washing processes, shown in Figure 9, created substantial pollutants that were emitted into air, water and the terrestrial environment. Moreover, the FETP indicator for geopolymer coating was mainly contributed by MK. Therefore, the MEP of geopolymer coating was higher than that of OPC coating. Other than these four indicators, which were dominated by either water glass or MK, geopolymer coating had significantly lower environmental impact potentials than OPC coating in Figure 10b. 

For the comparison between slag, MK and FA in Figure 11, slag and FA were concluded to be more eco-friendly than MK. The production of MK yielded intense impacts in terms of FDP, HTP, FETP, FEP and METP. The heating of kaolin at 1600 °C, displayed in Figure 9, possibly explained the high fossil depletion. The washing process of kaolin and MK emitted large amounts of wastewater into the water body, and the related indicators of FETP, FEP and METP were consequently increased, posing an imminent danger to human health. The environmental impacts of slag and FA in this study maybe overestimated due to the distorted system boundary in Figure 9. Both slag and FA were byproducts associated with other industrial activities. In this study, FA came from the hard coal combustion used for steel production, and slag was a byproduct of aluminum production. The adopted life-cycle inventory considered the related fossil fuel consumption, showing high GWP and FDP for slag and FA. The GWP and FDP of slag and FA would be greatly decreased if slag and FA were considered as recovered waste. In light of the potential overestimate, FA and slag were more eco-friendly than MK.

With the substitution of slag with MK, the overall environmental impact potentials generally increased when more MK was mixed in the geopolymer coating, as shown in Figure 12b. This finding was consistent with that shown in Figure 11b. When the weight of the other substances remained unchanged, more water was added, diluting the geopolymer. Given the same volume/FU of geopolymer coating, the overall environmental impact potential decreased proportionally with the increase in the W/S ratio.

## 5. Conclusions

This paper investigates the hydrophobic properties of geopolymer coatings using various surface modifiers. The contact angle, surface energy and critical surface energy were obtained. Two-phase contact angles between geopolymer and deionized water/eleven types of alkane were measured by the bubble method. A cradle-to-gate LCA was conducted to obtain the environmental impact of geopolymer coating, which was compared to that of OPC coating. The effects of different mixtures on the hydrophobic properties and environmental impacts were discussed. The following conclusions can be drawn from the test results and discussions:

(1) Similar to OPC coating, geopolymer coatings exhibited hydrophilic properties.
(2) Regardless of inner microstructure evolutions, the amount of water (the water/solids ratio ranges from 0.3 to 0.6), the properties of activators (water glass and NaOH), the constitutions of raw materials (except for the red mud) and ages (up to a year) for curing had negligible effects on the amounts and types of hydrophilic functional groups in geopolymer surfaces.
(3) Silicon-based modifiers exhibited better wetting performance than capillary crystalline surfactants. Silicon-based modifiers eliminated hydroxyl groups on geopolymer surfaces and maintained structural backbone Si-O-T (Si, Al).
(4) Immersion for 90 days in water without noticeable changes proved the stability of such modified geopolymer coatings.
(5) Geopolymer coating yielded substantially lower environmental impacts than OPC coating.
(6) Increasing W/S ratio diluted geopolymer coatings and decreased environmental impacts, and slag-based geopolymer coating achieved lower environmental impacts than the FA-based and MK-based varieties.

## Figures and Tables

**Table 1:** Chemical compositions of raw materials used in this research (wt. %).

	Chemical Oxides of Raw (wt. %)	SiO_2_	CaO	Al_2_O_3_	MgO	K_2_O	Na_2_O	Fe_2_O_3_	MnO	TiO_2_	SO_3_	SrO	ZrO_2_
Materials													
Slag		27.50	44.60	13.20	8.72	0.43	0.39	0.76	0.43	1.36	2.05	0.12	0.06
Red mud		16.36	2.95	26.49	0.41	0.19	11.33	36.01	0.06	5.01	0.51	0.04	
Metakaolin		53.12	0.08	42.21	0.18	0.55	0.47	2.38	0.03	0.56			
Fly ash		49.77	2.70	27.12	0.41	1.50	0.16	13.94	0.42	1.36	2.29	0.04	0.10

**Table 2:** Mix proportions.

Group	Specimen	Main Substance (g)				Activator (g)		Water (g)	W/S ^a^	Surface Modification	Curing Time (d)
		Slag	MK	Red Mud	Fly Ash	Sodium Silicate	NaOH Solution				
1	Reference-S0.4	300	0	0	0	168		36	0.4	- ^b^	28
	MK0.4		300			168		36	0.4	- ^b^	28
	RM0.4			300		168		36	0.4	- ^b^	28
	FA0.4				300	168		36	0.4	- ^b^	28
2	S0.3	300				168		0	0.3	- ^b^	28
	S0.5	300				168		72	0.5	- ^b^	28
	S0.6	300				168		108	0.6	- ^b^	28
3	75%S	225	75			168		36	0.4	- ^b^	28
	50%S	150	150			168		36	0.4	- ^b^	28
	25%S	75	225			168		36	0.4	- ^b^	28
4	S-WG1.4	300				148		55	0.4	- ^b^	28
	S-NaOH14	300					54	96	0.4	- ^b^	28
	S-NaOH10	300					71	79	0.4	- ^b^	28
5	S-56	300				168		36	0.4	- ^b^	56
	S-120	300				168		36	0.4	- ^b^	120
	S-360	300				168		36	0.4	- ^b^	360
6	S0.4-PTB	300	0	0	0	168		36	0.4	PTB	28
	S0.4-SY	300	0	0	0	168		36	0.4	SY	28
	S0.4-OSA	300	0	0	0	168		36	0.4	OSA	28
	S0.4-MSO	300	0	0	0	168		36	0.4	MSO	28

^a^ W/S: the ratio between the mass of water and solids. Water includes added water and those in water glass or NaOH solutions. ^b^: Not applicable.

**Table 3:** Measurement of surface energy and its components and work of adhesion of geopolymers at 22 ± 0.5 °C.

Sample Label	Dispersion Component (mN/m)	Polarity Component (mN/m)	Surface Energy (mN/m)	Work of Adhesion (mN/m)
Reference	21.56	44.72	66.28	138.87
MK0.4	22.08	44.94	67.02	139.63
RM0.4	22.48	47.13	69.61	142.33
FA0.4	20.71	45.87	66.58	139.23
S0.3	18.62	47.46	66.08	138.69
S0.5	21.54	44.77	66.31	138.91
S0.6	21.59	44.83	66.42	139.02
75%S	21.67	44.84	66.51	139.11
50%S	21.84	44.84	66.68	139.28
25%S	21.9	44.83	66.73	139.33
S-WG1.4	21.41	44.88	66.29	138.89
S-NaOH14	21.97	44.87	66.84	139.44
S-NaOH10	22.1	44.82	66.92	139.52
S-56	21.52	44.75	66.27	138.86
S-120	21.58	44.74	66.32	138.91
S-360	21.46	44.8	66.26	138.86

**Table 4:** Measurement of surface energy and its components and work of adhesion of modified geopolymers at 22 ± 0.5 °C.

Sample Label	Dispersion Component (mN/m)	Polarity Component (mN/m)	Surface Energy (mN/m)	Work of Adhesion (mN/m)
Reference	21.56	44.72	66.28	138.87
S0.4-PTB	23.1	8.5	31.6	86.52
S0.4-SY	29.62	9.73	39.35	95.37
S0.4-OSA	22.57	2.53	25.1	67.08
S0.4-MSO	14.68	6.54	21.22	72.3

**Table 5:** LCI analysis.

Substance	LCI	
	Source	Dataset
OPC	Gabi software	Professional database
FA	Gabi software	Extension database XIV: construction materials
MK	Gabi software	Professional database
Slag	Ecoinvent	Version 3.7
Water glass	Ecoinvent	Version 3.7
Deionized water	Gabi software	Professional database
OSA	Gabi software	Extension database XIV: construction materials

## Data Availability

Data available within the article.

## Appendix A

**Table A1:** Contact angles of geopolymers with various media.

Liquid	Contact Angle(°)									
	Reference	MK0.4	RM0.4	FA0.4	S0.3	S0.5	S0.6	75%S	50%S	25%S
Hexane	29	26.48	20.67	25.53	29.4	29	28.77	28.11	27.89	29.67
Heptane	29.33	27.48	19.67	25.08	28.48	29.33	29.41	29.1	29.11	28.45
Octane	28.31	27.45	23.2	25.35	29.33	28.31	29.33	28.79	29.13	28.36
Nonane	27.5	28.43	24.3	28.18	29.44	27.5	29.14	28.18	29.5	29.78
Decane	29.13	28.93	23.58	24.85	28.35	29.13	28.44	29.7	29.13	29.58
Undecane	30.65	31.93	22.45	26.08	30.1	30.65	27.99	29.08	28.65	28.98
Dodecane	30.01	29.78	23	26.65	28.99	30.01	28.59	28.65	28.55	29.01
Tridecane	30.6	29.55	24.73	27.98	30.11	30.6	29.15	27.98	28.98	28.44
Tetradecane	27.88	27	23.8	27.85	28.77	27.88	28.87	29.81	28.88	27.98
Hexadecane	29.01	28.7	20.93	23.08	29.7	29.01	29.14	29.04	29.01	29
Air	24.93	23.75	17.58	24.38	24.88	24.93	24.54	24.44	24.14	23.88

**Table A2:** Contact angles of geopolymers with various media (continued).

Liquid	Contact Angle(°)					
	S-WG1.4	S-NaOH14	S-NaOH10	S-56	S-120	S-360
Hexane	29.53	28.77	29.41	29.04	29.03	29.1
Heptane	29.08	28.45	28.67	29.21	29.3	29.39
Octane	29.35	28.31	28.97	28.4	28.37	28.07
Nonane	28.77	29.5	28.66	27.64	27.61	28.1
Decane	28.75	29.13	29.01	29.01	28.99	29.04
Undecane	28.08	29.11	29.44	30.45	30.65	29.89
Dodecane	27.88	29.01	29.58	29.47	29.9	29.42
Tridecane	27.98	28.7	29.01	30.12	30.33	29.87
Tetradecane	29.04	28.12	27.8	28.01	27.99	27.72
Hexadecane	29.14	29.12	29.02	29.14	28.78	28.73
Air	24.69	23.77	23.5	24.9	24.82	24.85

**Table A3:** Contact angles of geopolymers after modification with various media.

Liquid	Contact Angle(°)				
	Reference	S0.4-PTB	S0.4-SY	S0.4-OSA	S0.4-MSO
Hexane	29	105.3	98.5	118.8	105.75
Heptane	29.33	99.78	99.95	120.03	105.63
Octane	28.31	98.36	96.54	120	105
Nonane	27.5	101.54	95.47	119.53	105.5
Decane	29.13	100.23	97.88	122.3	106.1
Undecane	30.65	99.1	98.14	123.33	106.18
Dodecane	30.01	97.35	96.12	125.2	106.02
Tridecane	30.6	102.45	97.11	127.6	106.38
Tetradecane	27.88	103.45	99.7	127.8	106.88
Hexadecane	29.01	97.88	95.46	128.6	105.66
Air	24.93	78.78	71.45	95.63	90.75

## Notations

$W_{a}$ = Work of adhesion.

$\gamma_{1}$ = Surface energy of phase 1.

$\gamma_{2}$ = Surface energy of phase 2.

$\gamma_{12}$ = Interfacial energy between phase 1 and phase 2.

γ = The total surface energy.

$\gamma^{d}$ = The dispersive-force component of the surface energy.

$\gamma^{p}$ = The polarity-force components of the surface energy.

$W_{a}^{d}$ = Work of adhesion produced by the interaction of the dispersion force between molecules.

$W_{a}^{p}$ = Work of adhesion produced by the interaction of the polarity force between molecules.

$\gamma_{sw}$ = The solid–water interfacial surface tensions.

$\gamma_{si}$ = The solid–alkane interfacial surface tensions.

$\gamma_{wi}$ = The water–alkane interfacial surface tensions.

$\gamma_{i}$ = The alkane surface energy.

$\gamma_{i}^{d}$ = The dispersion-force component of the surface energy of the alkane.

θ = The equilibrium contact angle or eigen contact angle of the material.

$\gamma_{l}$ = The surface energy of the liquid.

$\gamma_{s}^{d}$ = The dispersion-force component of the surface energy of the solid.

$\gamma_{s}^{p}$ = The polarity-force component of the surface energy of the solid.

$\gamma_{l}^{d}$ = The dispersion-force component of the surface energy of the liquid.

$\gamma_{l}^{p}$ = The polarity-force component of the surface energy of the liquid.

$\omega_{l}^{d}$ = The proportions of the dispersion-force component of the liquid surface energy.

$\omega_{l}^{p}$ = The proportions of the polarity-force component of the liquid surface energy.

$\omega_{s}^{d}$ = The proportions of the dispersion-force component of the solid surface energy.

$\omega_{s}^{p}$ = The proportions of the polarity-force component of the solid surface energy.

$\gamma_{c}$ = The critical surface tension, referring to the characteristics of the solid surface.

Δγ = The energy difference between the surface energy of a given liquid and the critical surface energy of the solid.
